# Evaluation of Potential Transfer of the Pathogen *Saprolegnia parasitica* between Farmed Salmonids and Wild Fish

**DOI:** 10.3390/pathogens10080926

**Published:** 2021-07-22

**Authors:** Perla Tedesco, Marcia Saraiva, Jose Vladimir Sandoval-Sierra, Maria Letizia Fioravanti, Benedetto Morandi, Javier Dieguez-Uribeondo, Pieter van West, Roberta Galuppi

**Affiliations:** 1 Department of Veterinary Medical Sciences, Alma Mater Studiorum-University of Bologna, Ozzano Emilia, 40064 Bologna, Italy; marialeti.fioravanti@unibo.it (M.L.F.); benedetto.morandi2@unibo.it (B.M.); roberta.galuppi@unibo.it (R.G.); 2 Aberdeen Oomycete Laboratory, International Centre for Aquaculture Research and Development (ICARD), Institute of Medical Sciences, University of Aberdeen, Foresterhill, Aberdeen AB25 2ZD, UK; marcia.saraiva@abdn.ac.uk (M.S.); p.vanwest@abdn.ac.uk (P.v.W.); 3 CSIC—Real Jardin Botanico, 28006 Madrid, Spain; jsandoval@humboldt.org.co (J.V.S.-S.); dieguez@rjb.csic.es (J.D.-U.); 4 Instituto de Investigación de Recursos Biológicos Alexander von Humboldt, Bogotá 111311, Colombia

**Keywords:** *Saprolegnia parasitica*, salmonid farms, *Oncorhynchus mykiss*, *Salmo trutta*, *Salmo marmoratus*, *Salmo salar*, Italy, Scotland

## Abstract

*Saprolegnia* infections are among the main parasitic diseases affecting farmed salmonids. The distribution and potential transfer of *Saprolegnia* spp. between farms and the natural environment has been scarcely investigated. Therefore, this work aimed to study the diversity and abundance of oomycete species in salmonid farms, tributary water, and effluent water systems. Four trout farms in Italy and two Atlantic salmon farms in Scotland were considered. In Italian farms, 532 isolates of oomycetes were obtained from fish and water, at upstream, inside, and downstream the farms. In Scottish farms, 201 oomycetes isolates were obtained from water outside the farm and from fish and water inside the farming units. Isolates were identified to the species level through amplification and sequencing of the ITS rDNA region. In Italy, *S. parasitica* was significantly more present in farmed than in wild fish, while in water it was more frequently isolated from the wild, particularly in effluent systems, not associated with more frequent isolation of *S. parasitica* in wild fish downstream the farm. In Scotland, *S. parasitica* was the most prevalent species isolated from fish, while isolates from water were mostly *Pythium* spp. with few *S. parasitica* isolates from upstream and downstream the farms.

## 1. Introduction

Parasites of wild fish pose a potential threat to aquaculture [1]. One of the main issues related to intensive aquaculture is the proliferation of parasites, particularly those with direct life cycles, and other infectious agents due to high farming densities [2], which can subsequently spread outside the farm into the natural environment. Therefore, the risk of diseases spreading from wild to farmed fish, with subsequent proliferation within the farm and transmission into the environment, generates great concern.

In salmonid aquaculture, severe problems with respect to parasite exchange between farmed and wild fish have been reported [3,4,5]. Salmonid farming is mainly based on open aquaculture systems (open-net pens, raceways), which exchange water together with other materials, such as chemicals, waste, and a wide range of potential infectious agents with the natural environment (sea, natural streams).

Many oomycetes seem to be ubiquitous in freshwater environments, where they contribute to the structural and functional organization of aquatic ecosystems [6]. However, the introduction of potentially pathogenic oomycete species into lakes and ponds through fish stocking and other anthropogenic activities has been associated with the decline of amphibian [7] and crustacean [8,9] populations.

Infections with oomycetes of the genus *Saprolegnia* represent one of the main parasitic diseases affecting freshwater-farmed salmonids. *Saprolegnia parasitica* is of primary importance with respect to infections in fish, while other *Saprolegnia* species such as *S. diclina* and *S. australis* infect fish and their eggs [10,11,12,13]. *Saprolegnia* species can cause heavy losses in salmonid farming [10,14] and are responsible for the ‘winter kill’ in catfish aquaculture [15]. Although infections are usually more severe in farmed fish than in wild fish [16], *Saprolegnia* outbreaks have also been reported in the latter [17,18,19,20]. Particularly, high mortality rates were reported in wild populations of brown trout, *Salmo trutta*, in Spanish rivers [18]. This evidence raised concern about the possible spread of potentially pathogenic *Saprolegnia* strains from farmed fish to wild populations.

Despite the widespread occurrence of *Saprolegnia* spp. in wild and aquaculture environments [21], little attention has been directed to understand the distribution and potential transfer of these agents between the farm and its tributary and effluent systems. A recent study [22] on the distribution of *Saprolegnia* in selected trout farms in Croatia indicated a possible role of trout farms as a source of spreading *Saprolegnia* spp. into the environment. 

Until recently, difficulties in identifying oomycetes up to species level with morphological methods contributed to the lack of knowledge concerning their circulation in different farmed and wild fish [13,23]. The use of molecular techniques, based on amplification and sequencing of the ITS region, and phylogenetic approaches allowed to resolve problems in the identification of oomycetes and to establish DNA-based molecular operational taxonomic units (MOTU) for species delimitation [13].

The aim of this work is to study the distribution and species composition of oomycete assemblage through molecular identification of strains isolated in two different types of salmonid breeding and in their tributary and effluent water systems (trout farms located in Italy and Atlantic salmon farms located in Scotland), in order to: (i) study the diversity and abundance of oomycete species; (ii) evaluate the potential transfer of pathogenic species of *Saprolegnia*, specifically *Saprolegnia parasitica*, between wild and farmed fish, and (iii) determine whether there is an impact of salmonid farms on the diffusion of *S. parasitica* to wild fish in two different types of fish farming.

## 2. Results

Due to differences in the farmed species and the farming system/environment, the results will be described separately for Italy and Scotland.

### 2.1. Environmental Parameters

**Figure 1:**
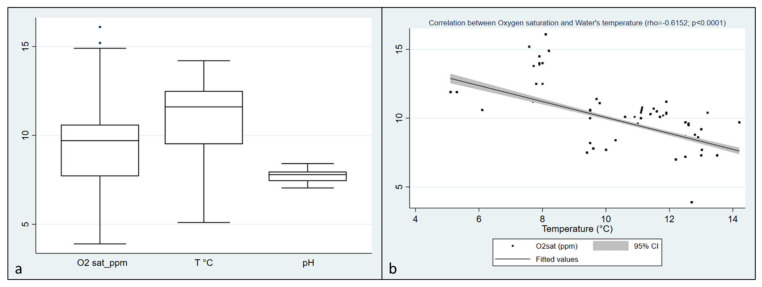
(**a**) distribution of Dissolved Oxygen, temperature, and pH values collected during the visits in Italian farms; (**b**) Correlation between Oxygen saturation and water temperature in Italian farms (rho = 0.6152, *p* < 0.0001).

In Italian trout farms, recorded values of Dissolved Oxygen (DO) ranged from 3.9 to 16.8, with a median of 9.7 ppm (IQRs: 7.7–10.6), water temperatures ranged from a minimum of 5.1 to a maximum of 14.2 °C with a median of 11.6 °C (IQRs: 9.5–12.5). Finally, the pH observation revealed a median of 7.79, ranging from 7.05 to 8.41 (IQRs: 7.43–7.96). (Table 1; Figure 1a). A negative correlation was found between Oxygen saturation and water’s temperature (rho = 0.6152, *p* < 0.0001) (Figure 1b). From the 239 collected fish, 458 single inocula were obtained, and 120 oomycetes strains were isolated. Additionally, 412 isolates were obtained from baits. 

**Figure 2:**
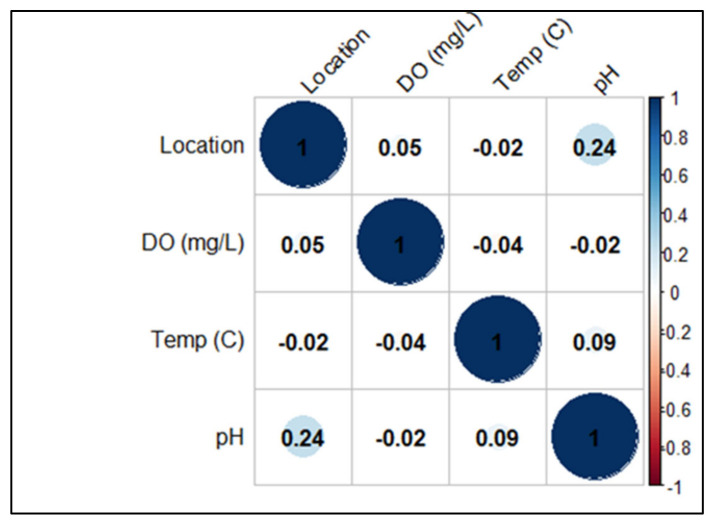
Pearson’s correlation coefficients calculated considering environmental data from Scottish farms.

In Scottish salmon farms, water temperature values ranged from 6.1 to 16.7 °C with a median of 10.3 °C (IQRs: 7.3–14.3), pH ranged from 4.5 to 7.9 with a median of 6.9 (IQRs: 6.6–7.2), being more constant at farm G (6.5–7.6), and dissolved oxygen values ranged from 90 to 94 mg/L (IQRs: 91–93) with a median of 92mg/L (Table 2). A negative correlation was found between DO and water temperature (*p* = 0.04) and between DO and pH (*p* = 0.02) (Figure 2).

From the 200 fish samples collected, 128 oomycetes were obtained. Moreover, 73 oomycetes were collected from hempseeds. 

### 2.2. Oomycetes Diversity

**Figure 3:**
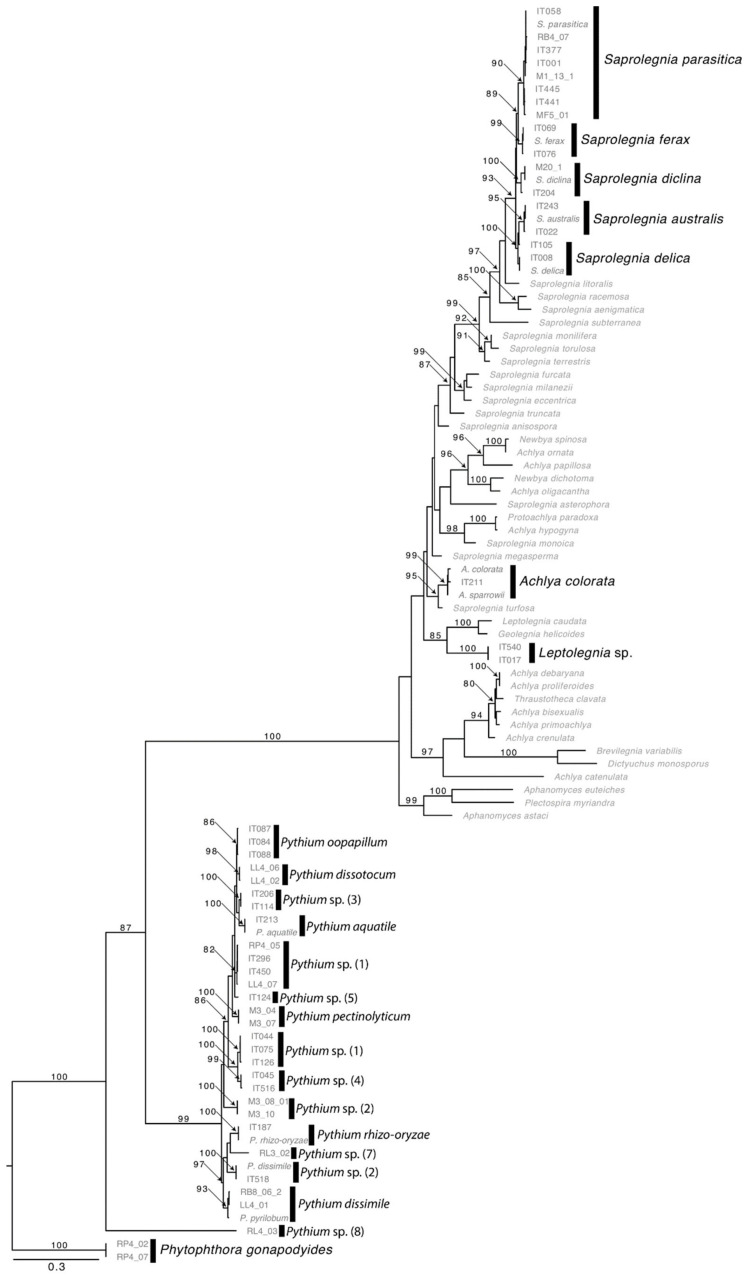
Maximum Likelihood tree based on ITS nrDNA sequences of oomycetes isolated during the present study with indication of the MOTUs (in bold) identified.

A total of 532 oomycetes isolates from Italy, and 201 from Scotland were identified by phylogenetic and molecular taxonomic analyses; the sequences obtained corresponded to the following MOTUs (Figure 3): *Achlya colorata*, *Saprolegnia australis*, *Saprolegnia delica*, *Saprolegnia diclina*, *Saprolegnia ferax*, *Saprolegnia parasitica*, *Pythium aquatile*, *Pythium dissimile, Pythium rhizooryzae*, *P. dissotocum, P. oopapillum*, *P. pectinolyticum* and other could only be identified to genus level, e.g., *Saprolegnia* sp., *Leptolegnia* sp., *Pythium* sp. 

**Figure 4:**
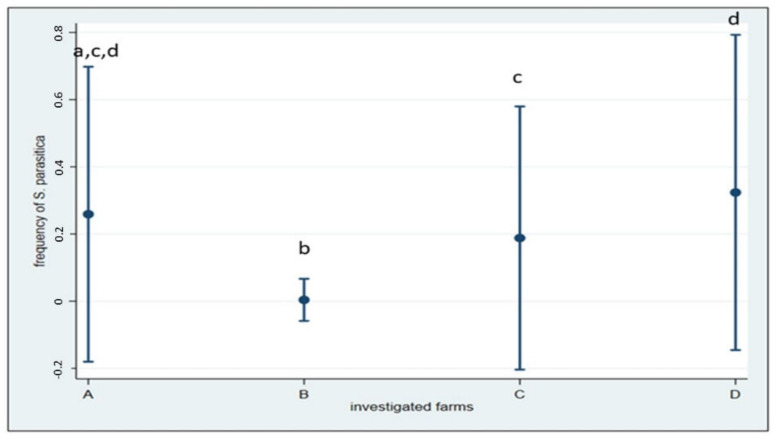
Frequencies of *S. parasitica* detected within the four Italian farms and the relative 95% CIs. Pairwise comparisons showed differences between farm A and B; B and C and B and D (Fischer’s exact *p* < 0.001), and between farm C and D (*p* = 0.002). Different letters indicate significant difference.

In Italian farms and connected aquatic systems, *S. parasitica* was isolated from 156 samples (17.9%). The frequency of *S. parasitica*, both from fish and water, was significantly different among the four surveyed farms (including their surrounding waters) (χ^2^(3) = 87.58; *p* < 0.001). Detection proportion ranged from 0.39% (1 positive sample) in farm B to a maximum of 32.4% (58 positive samples) in farm D. In particular, in farm B, where saprolegniosis is not considered a significant problem in farmed *O. mykiss*, *S. parasitica* was not isolated from fish upstream and inside the farm, but only from a wild *S. trutta* without lesions collected about 1 km downstream. Figure 4 shows the distribution of frequencies by farm.

DO did not significantly affect the probability of detecting *S. parasitica*. On the contrary, it was more frequently detected when water temperatures were colder (H = 10.48; *p* = 0.001) and with higher pH values (H = 45.58; *p* < 0.001). Quantitative analysis showed that there was 13% more chance to isolate *S. parasitica* for each Celsius degree decreased (*p* = 0.001).

**Figure 5:**
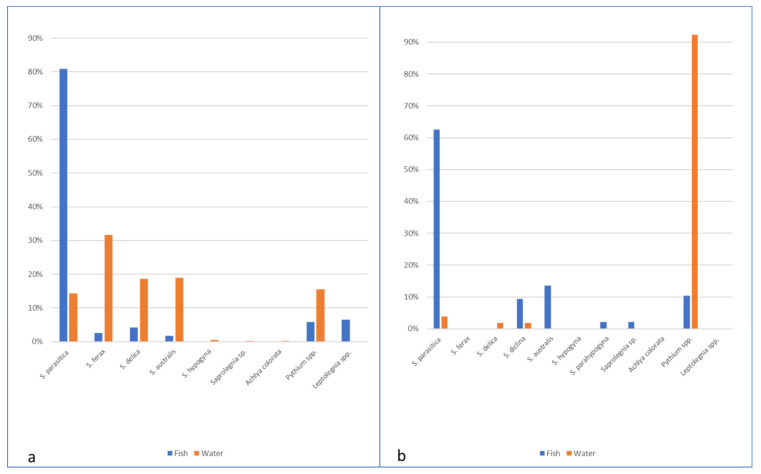
Species composition of oomycetes isolated from fish and water in Italian (**a**) and Scottish (**b**) farms.

*S. parasitica* was significantly more frequently isolated from fish (21.2% of the samples) compared to 14.3% of isolates from water baits (χ^2^ = 6.933, *p* = 0.008). Indeed, *S. parasitica* represented 80.8% of the oomycetes isolates from fish, while other species of oomycetes (e.g., *S. ferax*, *S. delica,* and *S. australis*) were more rarely found from different fish species (*O. mykiss*, *S. trutta,* and *Sq. cephalus*) alone or in association with *S. parasitica*. In eight farmed and wild fish (*O. mykiss*, *S. trutta*, *Sq. cephalus,* and *P. fluviatilis*), species of the genus *Pythium* were obtained. Particularly, in one wild *S. trutta* sampled downstream the farm, *Pythium* sp. was isolated from fin lesions. Isolates identified as *Leptolegnia* sp. were obtained in wild fish without lesions (1 *O. mykiss*, 4 *S. trutta,* and 1 *S. cephalus*) sampled downstream in one of the monitored farms. Using baits, a more heterogeneous variety of species was isolated (either from farm, upstream, and downstream water), and *S. ferax* was predominant (31.6% of the isolates), followed by *S. delica* and *S. australis*, while *S. parasitica* represented only 14.3% of the isolates). In particular, in farm B, *Pythium* spp. and *S. ferax* seemed to be prevalent in the aquatic environment, and *Pythium* sp. was also isolated from fish without lesions (2 farmed *O. mykiss*, 3 *S. cephalus,* and 1 *P. fluviatilis* downstream the farm) (Table 3; Figure 5a).

In Scottish farms and connected aquatic systems, *S. parasitica* was significantly more frequently isolated from fish compared to isolates from water baits (*p* < 0.0001). Indeed, *S. parasitica* represented 62.5% of the oomycetes isolates from fish, while other species of oomycetes, such as *S. diclina* or *S. australis,* were less found (9.3% and 13.54%, respectively). *Pythium* spp. were also found in fish (10.4%). In contrast with the Italian farms, no *S. delica* or *S. ferax* were isolated from fish. Farm F, although without reported saprolegniosis problems, presented a statistically significantly higher number of *S. parasitica* isolates (41) when compared to Farm G (20) with *p* = 0.006. This is probably the result of an intensive treatment regime from Farm G. In addition, the species distribution pattern was different between farms, with unique isolates of *Saprolegnia* sp1 being isolated from farm F and unique *S. delica* and *S. parahypogyna* isolates from farm G. Furthermore, a significant number of *Pythium* spp. isolates was obtained at farm G compared to farm F. In water, a diversity of *Pythium* spp. was isolated, representing 92.30%, followed by *S. parasitica*, *S. delica* and *S. diclina*. A reduced number of isolates was obtained at fish farms with only *Pythium* spp.; this is possibly due to the treatments carried out at the farm and the daily removal of mort’s. Upstream the fish farms, the only *Saprolegnia* species isolated was *S. parasitica,* while downstream only *S. delica* and *S. diclina* were isolated (Figure 5b, Table 4). No statistically significant difference was found between the water collection sites.

### 2.3. Transfer of Saprolegnia parasitica between Wild and Farmed Fish and Impact of Salmonid Farms on the Spread of S. parasitica to Wild Fish

**Figure 6:**
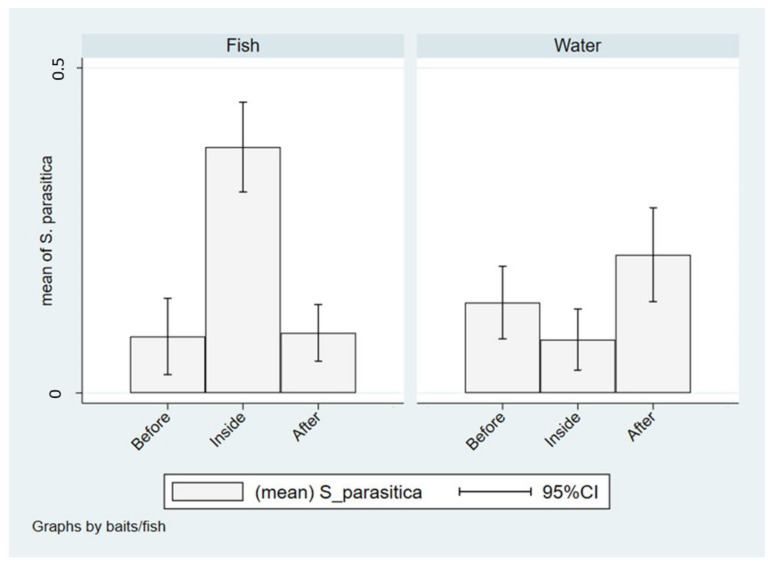
Isolation of *S. parasitica* in fish and water from upstream (before), in-farm (inside) or downstream (after) sampling sites in Italy.

In Italian farms, the isolation of *S. parasitica* was significantly higher in farmed fish than in wild fish from both upstream and downstream waters (χ2(2) = 55.37, *p* < 0.001), while no differences were found between wild fish captured upstream and downstream. On the contrary, the presence of *S. parasitica* in water, assessed through the use of baits, was significantly lower in farms compared to outside (χ^2^(2) = 9.085; *p* = 0.011) with particular references to downstream (Figure 6). 

The quantitative analysis remarks these differences, showing six times less chance to isolate *S. parasitica* in wild fish from upstream and downstream compared to farmed fish (*p* < 0.001), while when the isolation was made from waters using baits, it was three times more likely to detect *S. parasitica* downstream compared to in-farm (OR: 3.02; *p* = 0.004), whereas no significant difference was found when in-farm and upstream were considered (*p* = 0.132). These results are also summarized in Table 5.

## 3. Discussion

### 3.1. Oomycetes Diversity

Overall, a high number of oomycete isolates were obtained from fish and baits in the Italian and Scottish farms under study. 

Particularly, for Italian farms, the results obtained by culture/isolation of Oomycetes from water showed that hempseed baits allow the isolation of *Saprolegnia* spp., including *S. parasitica*, and other oomycete species from water in a very efficient way. Although *S. parasitica* is more frequently isolated from fish, our findings highlight the usefulness of environmental baits to isolate *Saprolegnia* spp. and other oomycetes during epidemiological studies, allowing to reduce the sampling of fish. Besides representing a useful tool to avoid invasive techniques in fish, the use of hempseed baits allows overcoming problems deriving from difficulties in sampling wild fish. For example, in Italy, in the upstream water system of farm B, only a few specimens of protected fish species were present; in farm A, in rainy periods the use of electrofishing was not feasible, and angling is difficult; in farm C, that receives the water from a spring, very few wild fish were present upstream. 

*Saprolegnia* species other than *S. parasitica* isolated in the present study (*S. delica*, *S. australis*, *S. ferax,* and *S. turfosa*), mainly through the use of baits, are often considered as part of the aquatic ecosystem and of lower importance for farming activities [21]. However, these species may also affect the aquatic fauna: particularly, *S. ferax* was involved, together with *S. diclina*, in mortalities of amphibian embryos [24]. Results of an experimental study [25] suggested that the transfer of *S. ferax* from restocked rainbow trout to wild amphibians was a possible cause of the decline of *Bufo boreas* populations, although few scientific data are available to support this hypothesis.

*Leptolegnia* species are often isolated from arthropods exuviae or from mosquito larvae [26]. Interestingly, in the present study, *Leptolegnia* was only found in six wild fish collected downstream the farm A; its isolation might be linked to the abundant presence of amphipod crustaceans in this environment. These amphipods are intermediate hosts of the swim-bladder nematode *Cystidicola farionis* and several acanthocephalans, parasites often encountered in trout cultured in farm A and may also be vectors of *Leptolegnia* spp. for other crustaceans and fish. However, no lesions associated with *Leptolegnia* were detected in the six positive fish.

Among all the farms taken into consideration in this survey, farm B was unique since it has a constant temperature of around 12 °C throughout the year, since the water originates from a brook approximately 600 m long, with particular environmental conditions (shallow water, constant temperature, abundant presence of algae, and low amount of dissolved oxygen), populated mainly by crayfish. The species composition of the oomycete assemblage may also be peculiar to this specific environment since, in this farm, a higher proportion of *Pythium* spp. was isolated. This genus, included within the order Peronosporales, comprises more than 200 species that are ubiquitous in soil or represent important plant pathogens [27]. However, they are also found in the aquatic environment [6], where they are isolated from animal and vegetal organisms. Particularly, several species of *Pythium* are found growing on dead specimens of freshwater crustaceans, where they contribute to the decomposition of chitin carapaces [28].

Both Scottish Atlantic salmon farms used in this study are loch sites with more than 60 m depth. Both areas are predominantly rural with moorland ridges, rocky outcrops, mosaics of heather, and grass with woodland, including forest plantations on some mid and lower slopes. Loch temperatures tend to be warmer in winter and colder in summer than adjacent rivers, showing the greatest variability in summer, taking longer to respond to seasonal temperature changes and rainfall events due to larger water volume. Lochs also have slightly acidic waters due to underlying geology. Due to all these adjacent conditions and *Pythium* spp. lifestyle (as mentioned above), the probability of isolating it from the water is high. Nevertheless, a variety of *Pythium* spp. were also isolated from fish. Its role in fish infection is unknown. Unique isolates of *Saprolegnia* sp1 were isolated from farm F, and unique *S. delica* and *S. parahypogyna* isolates from farm G. This might be due to host stage specificity of these pathogens since farm F possessed smolts, while farm G possessed parr and smolts. Interestingly, on farm F, which does not have recurrent saprolegniosis issues but treats fish prophylactically, a higher number of *S. parasitica* isolates were obtained from fish compared to farm G. Moreover, within the farms, *S. parasitica* was isolated almost exclusively from fish and not from water. At downstream sites, not many oomycetes’ isolates were obtained. Again, this might be due to the recurrent water treatment against saprolegniosis.

### 3.2. Transfer of Saprolegnia parasitica between Wild and Farmed Fish and Impact of Salmonid Farms on the Spread of S. parasitica to Wild Fish

Concerning the assessment of potential transfer and impact of salmonid farms on the spread of *Saprolegnia* to wild fish, the evaluation of the data collected in Italy must consider that the frequency of *S. parasitica* isolation, from both fish and water, was significantly different among the four surveyed farms, highlighting how the different environmental and managerial conditions can influence the presence and multiplication of this oomycete. A careful interpretation of the results of this survey should consider potential biases associated with the fact that several wild fish sampled upstream and downstream of the farms could have been introduced for restocking from other farms. In particular, farm A has affluent and effluent water within an irrigation channel system of Brenta River, where restocking with the allochthonous species *O. mykiss* is allowed because the channels are periodically drained and emptied. In this farm, although *S. parasitica* has always been isolated from cultured fish, it has never been isolated from wild fish downstream the farm but only from baits. It was isolated upstream from one “wild” *O. mykiss* with lesions that had been caught just before the grids of the farm. With respect to farm B, only one wild *S. trutta* without lesions was found positive to *S. parasitica* at about 1 km downstream the farm, in a channel where fish are often introduced for restocking. *S. parasitica* has never been isolated from *O. mykiss* in farm B, and no problems have been reported by the farm manager for several years. Concerning the sampling sites C and D, when *S. parasitica* was isolated from farmed fish, it was also always isolated from downstream fish. Upstream from these farms, *Saprolegnia* presence was almost always detected mainly through the hempseed baits, as some difficulties in sampling wild fish were often encountered at these sites. Nevertheless, the wild fish positive for *S. parasitica,* which were collected upstream and downstream the farms in most cases, were without lesions, except for one wild *S. trutta* upstream and one *S. trutta* downstream farm C and six wild *S. trutta* downstream farm D, which showed lesions with *S. parasitica*.

An interesting fact emerges from the statistical analysis of overall isolates obtained from Italian farms: *S. parasitica* was significantly more isolated in farmed fish than in wild fish, without differences between upstream and downstream sites; this is in accordance with the general principle that higher density of fish and other stressful factors associated with the farming environment may increase the colonization of *S. parasitica* in the fish host [29,30,31]. On the contrary, the presence of *S. parasitica* in water, determined by using baits, was significantly higher in the wild than within the farm and was particularly higher in downstream waters, although differences with upstream waters were not significant. Such finding would suggest that, with respect to the production systems surveyed in Italy, the farming of fish exhibiting overt signs of saprolegniosis could increase the dispersal of spores in water; on the other hand, this phenomenon does not seem to negatively affect wild fish populations, possibly due to the lack of potential predisposing factors (e.g., high stocking density, fish handling). 

In Scotland, the frequency of *S. parasitica* isolation, from both fish and water, was significantly different among the two surveyed farms, highlighting how the different handling conditions can influence the presence and multiplication of this oomycete, namely treatment regime, and handling associated with it. Although farm G had recurrent saprolegniosis issues, only a few *Saprolegnia* spp. isolates were obtained downstream of it. *S. parasitica* seemed to be concentrated on the fish itself, suggesting that this species is attracted to Atlantic salmon, a more susceptible species to saprolegniosis than trout, and due to high fish densities and fish stressors, it multiplies. Since no wild fish was sampled, due to conservation restrictions, we cannot make conclusions regarding the possibility of *S. parasitica* spread from farmed to wild fish. Nevertheless, no significant difference was found between samples collected from the different water sites.

## 4. Materials and Methods

### 4.1. Study Areas

**Figure 7:**
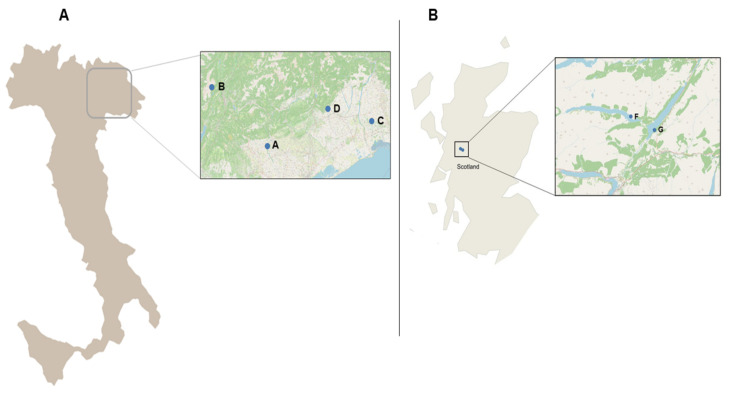
Farms considered for the study. (**A**) Farms’ location in Italy, (**B**) Farms’ location in Scotland.

For the part of the study carried out in Italy, four trout farms were considered (Figure 7A). In these farms, fish is cultured in concrete tanks in raceway systems that collect water from natural water bodies (river, spring, mixed water source) and discharge untreated effluents back into the environment. Cultured species include the rainbow trout *Oncorhynchus mykiss* (RBT), the brown trout *Salmo trutta* (BT), and the marble trout *Salmo marmoratus* (MT). 

For the part carried out in Scotland, two Atlantic salmon *Salmo salar* (AS) farms were considered (Figure 7B). In these farms, fish is cultured in an open-net pens system. 

### 4.2. Collection of Fish and Water Samples

In Italian farms, sample collection was carried out in three periods: (i) November to December 2015, (ii) February to March 2016, and (iii) November to December 2016; these periods were selected due to the higher infection rates with *Saprolegnia* reported by fish farmers in the area. During each period, ten cultured fish, preferably with lesions referable to *Saprolegnia*, were collected in each farm, possibly from different tanks/earth ponds where affected batches were observed. At the same times, up to 10 wild fish upstream and downstream from each farm belonging to different species (RBT, BT, MT, *Squalius cephalus, Perca fluviatilis, Scardinius erythrophthalmus*) were collected by electrofishing or angling. Overall, 239 fish (46 from upstream, 105 from farms, 88 from downstream) were collected. During the first two sampling periods in each tank/earth pond or water system, upstream, inside, and downstream the farm where fish were sampled, five traps (homemade tea balls) were placed, each containing seven sterile halves of boiled hempseed as baits (prepared according to Seymour [32]). The traps were left for at least 10 days and then retrieved. In total, 412 baits (151 from upstream, 134 from farm, 127 from downstream) were collected and used for oomycetes isolation. Information about Dissolved Oxygen (DO) expressed in part per million (ppm), pH, temperature (°C) of the waters was collected, before, within, and after the farms at the time of sampling and at the time of traps retrieval.

In Scotland, sampling of the water and farmed AS was carried out on a monthly basis in the two farms for 10 months (Feb-Nov 2016). During each sampling, ten fish per farm were collected randomly from inside the pen. Water samples were collected in 24-well plates with one hempseed on each well in each pen where fish were sampled and in the upstream (2 m before) and downstream (2 m after) water systems.

Water temperature, pH, and DO expressed in mg/L were recorded in each sampling location at the time of sampling.

### 4.3. Oomycetes Isolation

Oomycete isolates from Italy were obtained from clinically infected fish (two tufts of mycelia from two different fish lesions) from fish without lesions (a piece of gills and a piece of skin at the base of the dorsal fin) and from each hempseed bait. Each sample was dipped briefly in absolute ethylic alcohol, washed with sterile saline solution, and placed onto glucose-yeast extract agar supplemented with penicillin G (6 mg/L) and oxolinic acid (10 mg/L) [33] (GY+P+OX) plates. Isolates were incubated at 18 °C and re-cultured until pure cultures were obtained. 

A pectoral fin and skin close to the operculum were excised to obtain the Scottish fish isolates and placed into Potato Dextrose Agar (PDA) supplemented with ampicillin (500 mg/L), vancomycin (100 mg/L), and pimaricin (20 mg/L) incubated at 12 °C and re-cultured until pure cultures were obtained. The water samples were distributed in 24-well plates with hempseeds and incubated at 12 °C for 2 weeks or until visible growth was observed. Colonized hemp seeds were then placed into PDA supplemented media and incubated at 12 °C and re-cultured until pure cultures were obtained.

### 4.4. DNA Extraction and PCR Conditions

Single spore isolates from both Italy and Scotland were obtained in order to carry out DNA extraction and amplification, as described in Sandoval-Sierra and Diéguez-Uribeondo [23]. DNA extraction was carried out by using the DNeasy Plant Mini Kit (QIAGEN, Valencia, CA, USA). The internal transcribed spacer region (ITS) was amplified using universal primers for eukaryotes ITS5 and ITS4 [34] under the conditions illustrated in Sandoval-Sierra et al. [13]. Amplified products were sequenced using an automated sequencer (Applied Biosystems 3730xl DNA, Macrogen, The Netherlands). For each isolate, the consensus sequences for the ITS region were assembled and edited using the program Geneious v6.14 [35].

### 4.5. Molecular Identification of Species

For molecular identification of isolates to the species level, the ITS rDNA sequences obtained from all isolates were merged with *Saprolegnia* reference sequences according to Sandoval-Sierra et al. [13] using the software Geneious v10.0.9. The resulting sequences and *Saprolegnia* reference sequences were aligned using Mafft version 7.2 [36] and the algorithm G-INS-I [37,38]. The sequences were analyzed using the Maximum Likelihood model, and the isolates were assigned to species based on reference sequences for molecular operational taxonomic units (MOTUs) described by Sandoval-Sierra et al. [13]. Maximum Likelihood analysis was carried out using the software RaxML v8.1 [39]. For this analysis, the random starting tree was selected. Clade support was assessed with 1000 bootstrap replicates after selecting the best tree from 100 trees generated.

### 4.6. Statistical Analysis

In Italian farms where both fish and water samples were obtained from upstream, inside, and downstream sites, statistical analyses were carried out to evaluate the potential transfer of the pathogenic species *S. parasitica*, between wild and farmed fish and determine the potential impact of salmonid farms on the diffusion of *S. parasitica* to wild fish. The environmental data collected at the time of the visit and the results of oomycetes isolation and molecular identification were entered into a MS Excel spreadsheet and then imported into Stata 15 (StataCorp LLC, College Station, TX, USA) for statistical analyses. The distributions of environmental parameters were assessed by using the Shapiro–Wilk’s test for normality, median and interquartile ranges (IQRs) were reported as continuous variables were not normally distributed. Comparisons among *S. parasitica* isolates obtained from fish and baits in all the sampling sources (before, within, and after) and other categorical independent variables were computed by using Pearson’s χ^2^ considering the Fischer’s exact *p*-value when more than 20% of the cells had expected frequencies < 5 [40]. The Kruskal–Wallis non-parametric test was carried out when non-normally distributed continuous variables were involved. Odds Ratios (ORs) or inverse ORs (1/OR), when the OR is <1, and relative 95% Confidence Intervals (CIs) were also assessed to evaluate how the sampling sites, the sampling sources, and temperature affected the probability to isolate *S. parasitica*. The results were considered to be significant when *p* ≤ 0.05.

In Scottish farms, water samples were obtained from upstream, inside, and downstream sites, while fish were collected only from farms. The distributions of environmental parameters were assessed by using Shapiro–Wilk’s test for normality, median and interquartile ranges (IQRs) were reported as continuous variables were not normally distributed. Environmental parameters correlation was assessed by Pearson’s correlation using R. Comparisons among *S. parasitica* isolates obtained from fish and baits in all the sampling sources, and other categorical independent variables were computed by using Pearson’s χ^2^ using GraphPad. The Kruskal–Wallis non-parametric test was carried out when non-normally distributed continuous variables were involved.

## 5. Conclusions

Although genotyping of *S. parasitica* strains and other isolated oomycetes is necessary in order to assess their real circulation among upstream/farm/downstream systems, the results obtained in the present study seem to indicate that the interactions of *Saprolegnia* between wild and farmed fish are quite complex and might be influenced by anthropogenic interventions.

Specifically concerning *S. parasitica*, its occurrence seems mainly related to the presence of susceptible fish, as emerged from the results of isolations carried out in Italian farm B and the Scottish farms. When fish are present upstream of the farm, they can maintain the parasite in the environment and favor its spread to the farmed fish. In this regard, recommendations for reducing the transmission of oomycetes from wild fish to farms include avoiding the restocking in rivers with fish from infected farms. The typical conditions of salmonid farms, such as high biomass density, frequent handling of fish for grading and reproduction, and other predisposing factors, can favor *Saprolegnia* infection and pathology in the farmed population, with the multiplication of the parasite and increased release of spores into the wild. Therefore, although in our study we did not detect an effect of salmonid farms in increasing the spread of *Saprolegnia* to wild fish, such impact cannot be excluded, as pointed out also by Pavic et al. [22] in Croatia. 

In order to avoid a massive spread of infective stages of *Saprolegnia* spp. to the downstream water environment and wild populations, general recommendations to salmonid farmers should mainly rely on the application of good management practices aimed at reducing the oomycete load in the farm (e.g., early and frequent removal of clinically infected fish from the tanks/cages). 

The increased sustainability of fish farming activities and the protection of wild fish populations would benefit commercial and recreational fisheries. Particularly, a reduction in wild/farmed interactions would contribute to reducing the antagonism between wild fishery and aquaculture stakeholders emerging from the conflict of interests between different users. In this framework, the knowledge generated in the present study will inform the development of European policies aimed at protecting the health of wild aquatic animal populations while promoting responsible use of the aquatic environment for aquaculture purposes.

## Figures and Tables

**Table 1:** Characteristic of the farms considered in Italy and number of samples collected. Coordinates: A = 45°42′57.2” N, 11°40′49.2” E, 84 m a.s.l.; B = 46°32′644” N, 11°12′448” E, 250 m a.s.l; C = 45°54′53” N, 13°4′35” E, 30 m a.s.l.; D = 46°0′34” N, 12°29′38” E, 42 m a.s.l. farmed species: 1 = *Oncorhynchus mykiss*; 2 = *Salmo marmoratus* and *Salmo trutta* water supply: 3 = river; 4 = mixed (river/spring).

	Environmental Parameters (Min–Max)				n° Samples Collected			
Farm ID (Farmed Species/Water Supply	T (°C)	pH	O_2_ (mg/L)		Upstream	Farm	Downstream	Tot
A (1/3)	5.2–9.8	7.25–8.41	10–16.8	fish baits	20 34	30 32	16 38	66 105
B (1/3)	11.7–13	7.05–7.45	3.9–10.1	fish baits	1 38	30 45	42 30	73 113
C (2/4)	9.5–13.2	7.04–8.21	8.2–11.2	fish baits	15 39	25 27	20 35	60 101
D (2/3)	9.4–14.2	7.45–7.85	7.3–9.7	fish baits	10 39	20 30	10 24	40 93

**Table 2:** Characteristics of the farms considered in Scotland and number of samples collected. Coordinates: F = 56.966° N 5.134° W, G = 56°58′07″ N 4°54′38″ W; Farmed species: Atlantic salmon (*Salmo salar*).

	Environmental Parameters (Min–Max)				n° Samples Collected			
					upstream	farm	downstream	tot
Farm ID	T (°C)	pH	O_2_ (mg/L)					
F	6.1–16.7	4.5–7.9	91–93	fish baits	0 240	200 240	0 240	200 720
G	6.5–14.7	6.5–7.6	90–94	fish baits	0 240	200 240	0 240	200 720

**Table 3:** Oomycete isolates in Italy from water and fish samples.

Farm ID		n° Isolates Identified					Species Identified	
		Upstream	Farm	Downstream	Tot	Upstream	Farm	Downstream
A	fish	4	35	6	45	2 *S. parasitica* 1 *Pythium* sp. 1 *S delica*	31 *S. parasitica* 4 *S delica*	6 *Leptolegnia* sp.
	baits	35	32	38	105	1 *S. australis* 11 *S. parasitica* 13 *S. delica* 9 *Pythium* sp. 1 *S. ferax*	24 *S. delica* 6 *S. australis* 1 *S. ferax* 1 *S. parasitica*	13 *S. parasitica* 11 *S. delica* 7 *Pythium* sp. 4 *S. australis* 1 *S. ferax* 2 *S. hypogyna*
B	fish	0	2	5	7	0	2 *Pythium* sp.	3 *Pythium* sp. 1 *S. parasitica* 1 *S.ferax*
	baits	38	45	30	113	19 *Pythium* sp. 19 *S. ferax*	29 *S. ferax* 6 *Pythium* sp. 10 *S. delica*	18 *S. ferax* 4 *S. australis* 5 *Pythium* sp. 3 *S. delica*
C	fish	4	12	10	26	4 *S. parasitica*	12 *S. parasitica*	9 *S. parasitica* 1 *S. australis*
	baits	39	27	35	101	23 *S. ferax* 3 *S. parasitica* 6 *Pythium* sp. 5 *S. australis* 2 *S. delica*	6 *S. parasitica* 11 *S. ferax* 7 *S. australis* 2 *S. delica* 1 *Pythium* sp.	22 *S. australis* 7 *S. parasitica* 4 *Pythium* sp. 1 *S. delica* 1 *S. ferax*
D	fish	3	32	7	42	2 *S. parasitica* 1 *Pythium* sp.	30 *S. parasitica* 2 *S. ferax*	6 *S. parasitica* 1 *S. australis*
	baits	39	30	24	93	11 *S. australis* 7 *S. parasitica* 13 *S. ferax* 6 *Pythium* sp. 1 *Saprolegnia* sp. 1 *Achlya colorata*	11 *S. australis* 10 *S. ferax* 5 *S. delica* 4 *S. parasitica*	7 *S. parasitica* 7 *S. australis* *3 S. ferax* *6 S. delica* *1 Pythium* sp.

**Table 4:** Oomycete isolates in Scotland from water and fish samples.

Farm ID		n° Isolates Identified				Species Identified		
		Upstream	Farm	Downstream	Tot	Upstream	Farm	Downstream
F	fish	0	61	0	61	0	2 *P. dissotocum* 2 *P. flevoense* 2 *P. monospermum* 1 *P. pachycaule* 5 *S. australis* 6 *S. diclina* 41 *S. parasitica* 2 *Saprolegnia* sp1	0
	baits	14	8	18	40	2 *Phy. gonapodyides*	3 *P. dissotocum*	6 *P. dissotocum*
						7 *P. dissotocum*	2 *P. pachycaule*	1 *P. pachycaule*
						2 *P. pachycaule*	2 *P. pyrilobum*	7 *P. pyrilobum*
						2 *P. pyrilobum*	1 *S. parasitica*	2 *Pythium* sp03
						1 *S. parasitica*		1 *Pythium* sp05
								1 *S. parasitica*
G	fish	0	67	0	67	0	17 *P. dissotocum* 1 *P. flevoense* 1 *P. monospermum* 1 *P. pachycaule* 7 *P. pyrilobum* 1 *Pythium* sp01 2 *Pythium* sp03 1 *Pythium* sp05 1 *Pythium* sp08 8 *S. australis* 1 *S. delica* 4 *S. diclina* 2 *S. parahypogyna* 20 *S. parasitica.*	0
	baits	18	9	14	41	6 *P. dissotocum*	5 *P. dissotocum* 1 *P. pachycaule* 3 *P. pyrilobum*	10 *P. dissotocum* 1 *S. diclina* 1 *S. delica* 2 *Pythium* spp.
						1 *P. pachycaule*		
						7 *P. pyrilobum*		
						2 *Pythium* sp03		
						1 *Pythium* sp05		
						1 *S. parasitica*		

**Table 5:** Probability to isolate *S. parasitica* upstream or downstream the Italian farms compared to the in-farms baseline based on different sources and how the chance to isolate *S. parasitica* changes as a function of the water temperature. * Odds ratio is reported as the inverse (1/OR).

	Categories	OR	*p*-Value	95% CI
fish *	in-farm	*baseline*	-	-
	upstream	6.39	<0.001	2.92–13.95
	downstream	5.97	<0.001	3.31–10.78
baits	in-farm	*baseline*	-	-
	upstream	1.8	0.132	0.26–1.19
	downstream	3.02	0.004	1.43–6.39
T (°C)	-	1.13	0.001	1.05–1.21

In Scottish farms, the presence of *S. parasitica* in water, assessed using hempseeds as baits, was not statistically significant compared to upstream and downstream. Nevertheless, *S. parasitica* was isolated from farmed fish at the farms, with significant differences when compared to water (*p* = 0.0001). No wild Atlantic salmon was sampled due to conservation restrictions.

## Data Availability

The data presented in this study are available on request from the corresponding author.
